# Relationship between Maximum Force–Velocity Exertion and Swimming Performances among Four Strokes over Medium and Short Distances: The Stronger on Dry Land, the Faster in Water?

**DOI:** 10.3390/jfmk8010020

**Published:** 2023-02-01

**Authors:** Vincenzo Sorgente, Aaron Agudo-Ortega, Alejandro Lopez-Hernandez, Jesus Santos del Cerro, Diego Minciacchi, José María González Ravé

**Affiliations:** 1 Kinesiology and Motor Control (Ki.Mo.Co.) Laboratory, Department of Experimental and Clinical Medicine, Physiological Sciences Section, University of Florence, 50134 Florence, Italy; 2 Sport Training Laboratory, Faculty of Sports Sciences, University of Castilla-La Mancha, 45071 Toledo, Spain; 3 Castilla-la-Mancha Swimming Federation, Castilla-la-Mancha, 02001 Albacete, Spain

**Keywords:** sport science, sport performance, swimming performance, training prescription, strength training, exercise physiology

## Abstract

Evaluating force–velocity characteristics on dry-land is of the utmost importance in swimming, because higher levels of these bio-motor abilities positively affect in-water performance. However, the wide range of possible technical specializations presents an opportunity for a more categorized approach that has yet to be seized. Therefore, the aim of this study was to identify feasible differences in maximum force–velocity exertion based on swimmers’ stroke and distance specialization. To this scope, 96 young male swimmers competing at the regional level were divided into 12 groups, one for each stroke (butterfly, backstroke, breaststroke, and front crawl) and distance (50 m, 100 m, and 200 m). They performed two single pull-up tests, 5-min before and after competing in a federal swimming race. We assessed force (N) and velocity (m/s) exertion via linear encoder. There were no significant differences between pre-post maximum force–velocity exertions, despite the decreasing trend. Force-parameters highly correlated with each other and with the swimming performance time. Moreover, both force (t = −3.60, *p* < 0.001) and velocity (t = −3.90, *p* < 0.001) were significant predictors of swimming race time. Sprinters (both 50 m and 100 m) of all strokes could exert significantly higher force–velocity compared to 200 m swimmers (e.g., 0.96 ± 0.06 m/s performed by sprinters vs. 0.66 ± 0.03 m/s performed by 200 m swimmers). Moreover, breaststroke sprinters presented significantly lower force–velocity compared to sprinters specialized in the other strokes (e.g., 1047.83 ± 61.33 N performed by breaststroke sprinters vs. 1263.62 ± 161.23 N performed by butterfly sprinters). This study could provide the foundation for future research regarding the role of stroke and distance specializations in modeling swimmers’ force–velocity abilities, thus influencing paramount elements for specific training and improvement towards competitions.

## 1. Introduction

Swimming can be defined as a closed-skills sport, i.e., a sporting activity in which the environment is relatively highly consistent, predictable, and self-paced for performers [1]. Within this setting, the sole sport-specific practice cannot grant the swimmers a great enough stimulus to maximize their gestures [2,3,4]. Therefore, along with the acquisition of certain technical skills, the appropriate development of bio-motor abilities such as strength, power, and endurance are considered the key to success in competitive swimming [5,6]. In particular, strength can be described as the ability of the neuromuscular system to produce force against external resistance [7,8] and it has been repeatedly speculated to be the crucial bio-motor ability for the development of optimal swimming performance [9,10,11,12,13]. In this regard, some investigators advocate the paramount role of strength with a deduction: if higher levels of strength mean a higher capacity to produce force against water resistance, then consequently this will improve the swimmers’ velocity and ultimately their swimming performance [7,8,14,15]. Seeking to expand upon this compelling aspect, several scholars have in fact confirmed that there is a close relationship between the capacity of producing high forces and superior swimming performances. To this scope, a reliable tool used to assess swimming-specific strength (both on dry land as well as in the water) is the evaluation of force–velocity exertion, which basically dictates the relationship between the load lifted and the speed it can be moved [16,17]. Specifically, it has been shown that high levels of maximum strength in various exercises (e.g., bench press, pull-up, back squat, horizontal rows, etc.) correlate with many technical components of a swimming race, such as trunk stability during the stroke, gliding phase, diving phase, turning phase, stroke length, stroke frequency, and stroke index [2,3,4,6,7,11,18,19,20,21,22,23,24,25], ultimately translating into optimized swimming velocity [19]. Nonetheless, vertical pulling gestures are the most used and effective motions for evaluating force–velocity production in swimming performances [11,18]. In particular, it has been shown that the maximum velocity and force generated during the pull-up exercise highly correlates with swimming velocity [21]. These findings well demonstrate the effectiveness that well-developed bio-motor abilities, assessed through force–velocity parameters on dry land [20], hold on swimming performance.

However,, despite this promising body of research, the extensive heterogeneity lying within competitive swimming raises several issues that are yet to be ascertained. For instance, one critical element concerns the broad technical outlook, i.e., the four “cardinal” swimming strokes (butterfly, backstroke, breaststroke, and front crawl). Although most of the literature is focused on the front-crawl stroke [26,27], it is worth noting that the butterfly, backstroke, and breaststroke “styles” are highly specific as well as biomechanically and kinematically different from each other [28]. Based on the unique characteristics of each stroke, it could be that their differences are also reflected in the force–velocity exertion of specialized swimmers. If that is the case, it would then be possible to outline the ideal bio-motor features of swimmers who concentrate their endeavors on a particular stroke.

Another issue pertains to the numerous distances that are swum in swimming races, which dictate the athletes’ specialization from a bio-energetical standpoint. On this subject, suitable research has highlighted the diverse aerobic, anaerobic, and technical demands that reside among sprint and medium- and long-distance swimming races [26,29]. Nevertheless, it should be considered that medium-distance sporting activities (as a 200 m swimming race would be defined) also seem to benefit from the high capacities of producing muscular force [30,31,32]. Seeing these ambiguous findings, there is a need to clarify whether measures of force–velocity significantly change depending on different swimming distances, eventually drawing out the implications in terms of building superior in-water performances based on distance specialization.

As we argued in the paragraph above, when it comes to dissecting how force–velocity levels pertain to various swimming specialties during training, the current literature presents more questions than answers. Still, these uncertain aspects provide appealing opportunities for further inquiries. With these considerations in mind, in the present study, we sought to evaluate the relationship between maximum force–velocity exertion and swimming performances in male regional-level swimmers, examining and comparing both medium (200 m) as well as short (50 and 100 m) distances; all the four strokes swum in competitions; and the amount of neuromuscular fatigue generated by the competition, collecting force–velocity data shortly before and after the swimming race. The single pull-up test, performed for one repetition exerting maximum force, was used to evaluate the swimmers’ force–velocity production, whereas the official time of the respective swimming race (swum in a short course of 25 m) was considered as an indicator for swimming performance.

Ultimately, the purpose of this article is to clearly define to what extent maximum force–velocity capacities and swimming performances relate to each other when considering the intrinsically different bio-motor and technical facets of athletes specialized in a certain stroke and distance, also exploring whether and how this relationship is altered due to neuromuscular fatigue after performance. Overall, this additional knowledge would make it possible to draw evidence-based indications in order to further elevate swimming performance, favoring both academics for more profound investigations as well as coaches and athletes striving to attain ever-better competitive forms. 

## 2. Materials and Methods

### 2.1. Participants

A homogeneous group of 96 male swimmers competing at the regional level took part in the study (16 ± 1.3 years of age; height of 175 ± 2.7 cm; weight of 69 ± 2.2 kg; 6.5 ± 1.1 years of experience; 466 ± 21 Fédération Internationale De Natation points of best competitive performance). Specifically, we divided the subjects per stroke (i.e., butterfly, backstroke, breaststroke, and front-crawl) and distance (i.e., 50, 100, and 200 m), therefore assigning eight subjects per group. All of the participants reported no physical injuries prior to or during the duration of the study. Moreover, the subjects were already familiar with the pull-up motion from their previous training experience. The participants were all tested individually. All of the subjects provided assent and the parents/guardians provided informed consent after a detailed description of the study procedures. The study was approved by the local Ethics Committee of the university (FGM02102019) and was conducted in accordance with the Declaration of Helsinki.

### 2.2. Data Collection

We collected data regarding the in-water performances and the force–velocity parameters. As for the in-water performance, swimming race times were collected during federal swimming races by professional personnel employed in the local swimming federation. As for the force–velocity parameters in the ascending phase of the single pull-up test, velocity (m/s) and force (N) were collected using the Vitruve linear encoder (Speed4Lifts, Madrid, Spain). Specifically, this linear encoder comes in the portable form of an 8 cm^3^ box, equipped with an extensible wire that is attachable via a Velcro strap. Moreover, the Vitruve linear encoder is embedded with a smartphone app that allows for insertion of the subject’s height and weight, consequently calculating specific performances in a selected exercise (in this case, the pull-up). In particular, we used the velocity-based data registered by the encoder (i.e., power and velocity) to provide the force values.

### 2.3. Procedures

The subjects performed the single pull-up tests and concurrent swimming race at the end of their preparatory cycle of training (i.e., 8 weeks after the start of the season). The experiments were performed inside a regular, short-course (25 m) competitive swimming facility, during competition days. In particular, the single pull-up tests were performed in a large, quiet room inside the facility and near the pool. Furthermore, the pull-ups were performed using a standard steel bar of 3.81 cm in diameter (1.5 inches), standing 2.50 m from the ground.

Prior to the start of the experiment, the participants were advised to perform two single pull-up tests, with the first test taking place 5 min before the swimming competition and repeating the same test 5 min after the aforementioned competition. Specifically, the subjects were instructed to perform one repetition of the pull-up motion, exerting the highest force possible (i.e., pulling as strong and fast as they can). Moreover, the subjects had to follow precise criteria regarding the pull-up execution; first, they had to reach for the bar with a prone grip, without their feet touching the ground and by maintaining their arms and elbows straight. This was considered the starting position of the pull-up test. From this hanging position, after a brief verbal cue (“Ready, go”), the subject would then perform the pull-up, which had to be executed without any movement of the legs and passing with the chin over the bar. To ensure the procedure for measuring pull-ups 5 min before and 5 min after the swimming races, we employed a stopwatch. According to Kraemer and Fleck [33], 5 min is considered a long rest period, capable of dissipating the amount of fatigue experienced during anaerobic physical exertions. According to this information, we used the 5 min rest period to ensure enough recovery pre-competition and to also establish the same rest period after the competition.

In order to prepare for the competitions, the subjects first executed 20 min of warm-up. Specifically, the warm-up was 20 min long and consisted in the first part (about 5 min) being performed on dry land using body weight (e.g., squats) and elastic bands exercises (e.g., shoulders horizontal internal and external rotation), whereas the second part (about 15 min) was performed in-water and included sport-specific drills and exercises (i.e., turns, underwater glides, swimming at various paces and stroke rhythms), performed at light intensity. After 20 min of warm-up (i.e., 5 min before the respective swimming race), the subject came into the testing room. In order to register the force–velocity data, the linear encoder was attached to the subjects’ hips through a harness. The linear encoder was instead attached to the ground, within the same vertical plane as the subjects. In this way, it was possible to collect accurate data with minimal invasiveness. After that, the subjects performed the single pull-up test according to the criteria explained above. Five minutes after the single pull-up test, the subjects took part in the federal swimming race assigned. Each swimmer took part in only one race. Another five minutes after the swimming race, the subjects returned and performed a second single pull-up test following the same experimental setup. Each swimmer that was recruited for this study was tested individually for both the dry land and in-water performance assessments. However, given the competitive nature of this experimental setting, the subjects competed in the swimming races with other athletes who did not take part in this study.

### 2.4. Statistics

The data were analyzed using IBM SPSS Statistics 26 software. Parametric analyses were conducted as the Shapiro–Wilk test revealed a normal distribution of data (*p* > 0.05). First, we checked for test–retest reliability taking advantage of the two force–velocity assessments we made within this experimental setup. The resulting correlation coefficient was 0.81, therefore indicating an instance of good test–retest reliability. Then, detriments in the pull-up performance due to neuromuscular fatigue were sought using the ANOVA repeated measures test for both the velocity and the force generated before and after the swimming race. Furthermore, the Pearson correlation coefficient was used to define the levels of dependence among the force and the velocity in the single pull-up test and the time of the swimming races. Moreover, we used a multiple linear regression model to quantify the relationship between the velocity and force in the single pull-up test with the time of the swimming races. Finally, we implemented one-way ANOVA followed by the Bonferroni post-hoc test for multiple comparisons in order to detect differences in velocity and/or force exerted in the single pull-up test among strokes and distance specialties.

## 3. Results

The fatigue generated by the swimming performances affected both the velocity as well as the force in the single pull-up test (Table 1). Specifically, the pre-post percentage difference in velocity ranged from −1.71% in the 100 m backstroke group to −2.86% in the 50 m breaststroke group, whereas the percentage difference in force ranged from −2.43% in the 100 m breaststroke group to −3.39% in the 100 m backstroke group. However, the ANOVA repeated measures test reported no statistically significant differences in either velocity (F (96, 1) = 1.89, *p* = 0.17) and force (F (96, 1) = 2.33, *p* = 0.35) among the groups. While including a control group to test for fatigue after the swimming race would improve the experimental design, we have no reason to believe that the general loss in force–velocity post-competition was not due to the swimming race, which was the only physical stimulus occurring between the pre- and post-evaluations. Moreover, we employed more than enough recovery time regarding the single pull-up tests before and after the swimming race (i.e., 5 min) [33]. Consequently, it was reasonable to expect similar values of force–velocity performances from the subjects, which was only partially the case. However, we again specify that this trend did not achieve statistical significance, and thus can only be seen as a speculative interpretation of the phenomenon.

**Figure 1:**
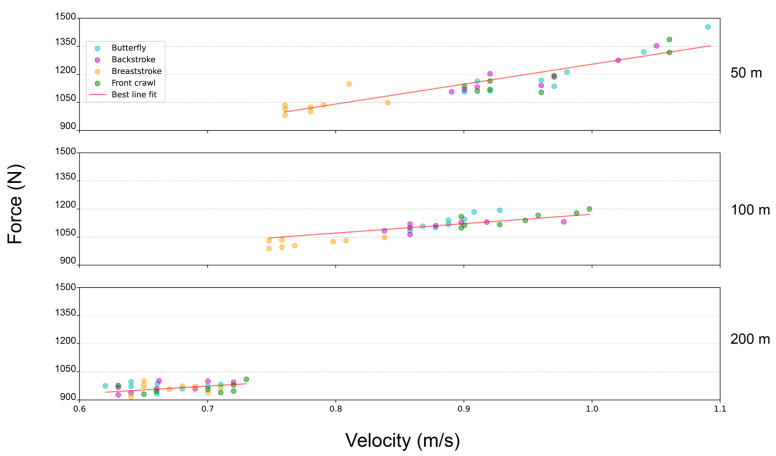
Scatter plot showing the maximum force–velocity exertion of the swimmers, grouped by stroke, and differentiated by swimming race distance. As shown by the best line fit, the high correlation between the force and velocity values in the single pull-up test reflects an almost linear trend. This is particularly evident within the 50 m and 100 m groups of swimmers.

Considering the whole sample of subjects (*n* = 96), the application of the Pearson r coefficient revealed a strong correlation between velocity and force (0.94 and 0.93 for the pull-up pre and post competition, respectively), suggesting that these two parameters may describe the same trend in this context. Likewise, the correlation between the swimming race time and force in the pull-up test was −0.74 both pre and post competition, whereas the correlation between the swimming race time and velocity during the pull-up test was −0.86 both pre and post competition, indicating that stronger/faster performances in the single pull-up test correlate with lower (thus better) swimming race times. Moreover, this strict correlation between force and velocity indicates that there is an almost linear relationship between them (i.e., more force generated means reaching a higher velocity and vice versa) (Figure 1).

Multiple linear regression was calculated to predict swimming race times based on the velocity and force generated in the single pull-up test, before the competition. In order to include all of the results in the same explanatory model, we standardized the swimming race times for each distance and stroke, calculating the respective z-scores. In a similar way, given the differences occurring in both force and velocity requirements in the single pull-up test among the experimental groups, the independent variables (i.e., velocity and force) were also standardized by z-scores. It is necessary to standardize the values since the explanatory variables in regression models have different scales and different levels of size. Considering the multiple linear regression analysis, a significant regression equation was found (F (2, 93) = 78.17, *p* < 0.001), with an R2 of 0.81 and an R2 adjusted of 0.80 (Table 2).

In particular, the swimmers’ predicted race time was equal to 3.03 × 10^−14^—0.046 (velocity)—0.037 (force). It should be noted that positive z-score values indicate values above the group mean; therefore, an increased z-score represents an increase in either the velocity or the force. Specifically, the model showed that a unitary increase in the velocity z-scores resulted in a decrease in the target variable (z-point, i.e., the swimming race time) of 0.046 s, whereas a unitary increase in the force z-scores predicted a slightly smaller decrement of 0.037 s. Bearing in mind that positive z-point values correspond to swimming race times above the mean and vice versa, the above behavior indicates that the higher the velocity or force generated by the athletes, the shorter their swimming race time. In addition, both the velocity (t = −3.90, *p* < 0.001) and force in the pull-up tests (t = −3.60, *p* < 0.001) were significant predictors of swimming race time.

Finally, the one-way ANOVA was performed to compare the differences in velocity and force based on stroke and distance (Table 3). Regarding the validation of the test, although the a priori power was low (0.28), the null hypothesis was still rejected. Moreover, the one-way ANOVA revealed that there was a statistically significant difference in both velocity (F (11, 84) = [66.80], *p* > 0.001) as well as force among the groups of swimmers (F (11, 84) = [19.60], *p* > 0.001).

Specifically, Table 4 reports the Bonferroni post-hoc test for multiple comparisons (Table 4). A fascinating aspect emerging from the post-hoc analysis is that almost all the significant differences between the groups in velocity corresponded to the same significant differences between groups in force, with the only exceptions being between the 200 m backstroke and the 50 m breaststroke (i.e., the mean difference in velocity was 0.12 ± 0.09 and statistically significant, whereas the mean difference in force was 87.01 ± 61.48 and not statistically significant) and between the 50 m breaststroke and 100 m front crawl (i.e., the mean difference in velocity was 0.15 ± 0.10 and not statistically significant, whereas the mean difference in force was 140.86 ± 99.54 and statistically significant). This further confirms the assumption that within this experimental setting, there is an almost linear relationship between maximum force and velocity productions.

**Figure 2:**
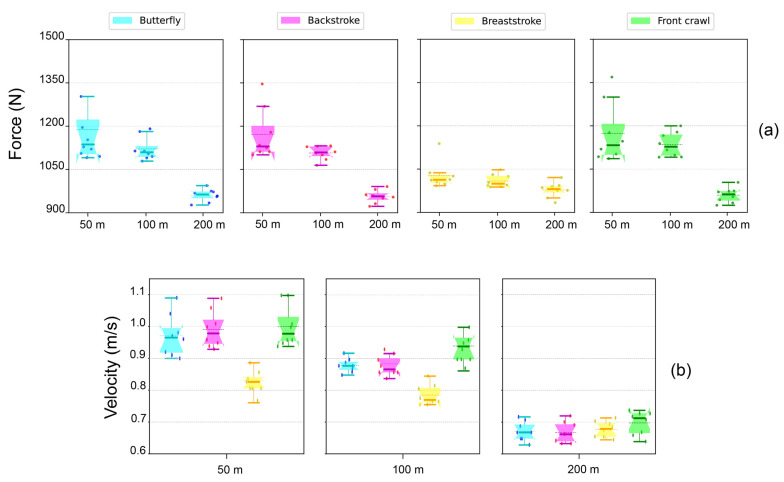
Scatter box plot showing the maximum force (N; panel (**a**)) and velocity (m/s; panel (**b**)) exerted in the single pull-up test, before the swimming competition. (**a**) Within all four strokes, a trend emerged where the sprinters (both 50 m and 100 m) could exert significantly higher forces compared to the medium-distance swimmers. Although it remained statistically significant, this trend was less evident regarding the breaststroke swimmers. (**b**) The 50 m and 100 m breaststroke swimmers presented significantly lower levels of velocity in the single pull-up test compared to the swimmers of other strokes competing in the same distance. However, this was not the case with the group of 200 m swimmers, where there were no significant differences among the groups.

When grouping the swimmers by stroke specialization, the 50–100 m sprinters were significantly faster and stronger in the single pull-up test than the 200 m middle-distance swimmers (Figure 2). Despite still being statistically significant, this tendency was quantitatively less prominent for the breaststroke performers. Instead, when categorizing the swimmers by the same race distance, we observed significant differences in maximum force–velocity exertion among breaststroke sprinters (i.e., 50 m and 100 m) and the other three strokes (i.e., butterfly, backstroke, and front crawl, both in 50 m as well as 100 m). Specifically, the 50 m and 100 m breaststroke performers presented significantly worse force–velocity parameters in the single pull-up test than their butterfly, backstroke, and front crawl peers (Figure 2). Regarding the 100 m swimmers, although there were no significant differences in force–velocity values compared to their 50 m counterparts, the force–velocity peaks were always reached by the 50 m sprinters. Furthermore, this behavior was not present in the middle-distance (i.e., 200 m) swimmers, which showed no significant differences from one stroke to another, although their maximum force–velocity exertions were all significantly lower compared to the 50 m and 100 m strokes.

## 4. Discussion

In the present study, we sought to explore the relationship between maximum force–velocity exertion and competitive performances in regional-level swimmers, specialized both for stroke (i.e., butterfly, backstroke, breaststroke, or front crawl) and race distance (i.e., 50, 100, or 200 m). The purpose of this investigation was to leverage a reliable and robust assessment method of the athletes’ bio-motor abilities in order to acknowledge trends, patterns, and differences capable of bringing valuable insights for highly specialized performance enhancements in swimming.

In previous review articles, it has been argued that more accomplished swimmers presented significantly lower energy expenditure, especially among swimmers specialized in middle-distance and long-distance races [15,28,34,35]. This was due to energy expenditure being a more limiting factor in swimming races from 400 m to 1500 m than neuromuscular-fatigue-related variables in 50 m, 100 m, and 200 m swimming races. According to the work of Pyne and Sharp [34], this implied that there could be a considerable effect of stroke and distance specialization on “swimming economy” and energy system management, which could be investigated by measuring neuromuscular energy expenditure in sprinters and middle-distance swimmers. Nevertheless, although our findings highlighted a trend of a general reduction in both velocity and force generated in the single pull-up test shortly after the swimming competition (Table 1), the ANOVA repeated measures revealed no significant differences between the pre- and post evaluations (F (96, 1) = 1.89, *p* = 0.17 concerning velocity and F (96, 1) = 2.33, *p* = 0.35 concerning force, respectively). Thus, in contrast with the above-mentioned measurement (i.e., VO2 max consumption), neuromuscular energy expenditure does not seem to be a specific enough method to assert either specific performance features or differences among stroke/distance specializations in swimming.

We found a strong correlation in the form of the Pearson r coefficient between force (N) and velocity (m/s) parameters in the ascending phase of the single pull-up test (i.e., 0.94 and 0.93 for the pull-ups pre and post competition, respectively). This outcome confirms the results of other recent investigations which have suggested concentrating dry-land training efforts on enhancing the neuromuscular abilities of swimmers, particularly on the integration and coordination of musculature to perform specific tasks under high loads or in an explosive fashion [16,22,28]. Similarly, the same analysis showed a high degree of correlation between the swimmers’ maximum force–velocity exertion and their respective race times (i.e., −0.74 between the force and swimming race time and −0.86 between the velocity and swimming race time). In this regard, we are in line with the research work conducted by Perez-Olea et al. [21], which showed that the 50 m front crawl swimming time was highly correlated with force–velocity variables of the ascending phase of the single pull-up test. Moreover, our multiple linear regression analysis further substantiates the validity of the pull-up motion mechanics to predict swimming performance in trained swimmers (Table 2). Hereof, the beta coefficients (i.e., velocity and force z-scores) were both negative. This means that the higher the value of these beta coefficients, the shorter the time in the swimming race, ultimately resulting in a better competitive outcome. These findings further promote the analysis of pull-up mechanics as a valid, efficient, and reliable means to both calibrate and predict crucial aspects of competitive swimming performances. Concerning this aspect, it is worth noting that we designated the swimming race times as a measure of in-water performance. However, it would be interesting to expand upon this research topic also considering more specific aspects that effectively contribute to the final performance. For instance, analyzing measures of technical proficiency such as stroke length, stroke index, stroke frequency, drag area during stroke, etc., could provide further support in understanding how maximum force–velocity exertion reshapes based on the swimmers’ stroke-distance specialization and how scholars and coaches could leverage these distinctions to enhance highly specific elements of swimming performance.

Among all the strokes, the 50 m and 100 m sprinters had significantly higher force–velocity values in the single pull-up test than the 200 m middle-distance swimmers (Table 4). Nonetheless, despite maintaining statistical significance, this trend was flattened for the breaststroke performers compared to the other three strokes (Figure 2).

In terms of swimming performance optimization, we confirm that the ability to produce higher amounts of force–velocity can indeed be useful in improving swimming race times, especially in sprinters [18,19,20,21,23,28,35]. However, our results also indicated that force–velocity values tended to be lower in competitors specialized in middle-distance races (Figure 2). In this regard, it is well established that the specific contributions of various energetic systems depend on both the length of the race and the intensity of the pace used [33]. Specifically, middle-distance competitors may prioritize the maximization of aerobic capacities in lieu of force–velocity abilities, which are more related to anaerobic capacity and neuromuscular factors [7,20,21,22]. Furthermore, this bio-energetic shift necessitates ulterior technical adjustments such as maintaining stroke efficiency (i.e., sustaining parameters of stroke length, stroke frequency, and stroke index) for a longer time compared to 50 m and 100 m swimming races [34,35]. The generally lower force–velocity values in middle-distance swimmers may be also favored by the greater configuration of technical parameters from a tactical–strategical perspective, which is less present in sprinting competitions [34].

Still, there are several reasons to advocate for a leveling up of maximum force–velocity levels even in middle-distance swimmers competing at the regional level. For instance, in the present study, we found a considerable correlation between higher productions of force–velocity and superior swimming performances, including the 200 m performers. Moreover, this is in line with several scholars who observed that underdeveloped levels of force–velocity can result in an early deterioration of technical skills due to the accumulation of neuromuscular fatigue [7]. These aspects would also definitely benefit the in-water performance of middle-distance swimmers. Therefore, we strongly encourage trainers to fill the apparent gap in bio-motor skills between sprinters and middle-distance swimmers, providing the latter with more focus and training time to upgrade their force–velocity capacities.

In addition, possible alterations in maximum force–velocity production due to specific training periods should be considered [5]. For instance, in this study, we collected force–velocity data at the end of the swimmers’ preparatory cycle of training (i.e., after the first 8 weeks of training). However, considering both the differences in training as well as the significant gap in bio-motor abilities that we found between sprinters and 200 m performers, it may be that the force–velocity capacities of middle-distance swimmers are greatly susceptible to the variations in training intensity and volume occurring over the season (e.g., from the preparatory cycle of training to the competitive cycle of training). For these reasons, we recommend future studies to carefully analyze hypothetical fluctuations in swimmers’ force–velocity levels over a competitive season and how these fluctuations may affect swimming performances, especially for middle-distance swimmers. 

Notably, we found a significant gap in maximum force–velocity production in breaststroke sprinters compared to the other 50 m and 100 m strokes (Figure 2). Indeed, we should account for some technical and biomechanical restraints regarding stroke velocity and general efficiency in breaststrokes, especially compared to the butterfly, backstroke, and front-crawl styles of swimming. Here, the basic assumption is that in order to reach, maintain, and increase in-water velocity, swimmers must continuously generate muscular propulsive forces to “fight” and exceed the drag forces of water. However, it is worth mentioning that breaststroke swimming produces the largest intracycle velocity variability among the four strokes [34]. This is due to the added drag of recovering both arms under the water and in drawing the knees up to prepare for the next propulsive phase of the stroke cycle. In fact, breaststroke is the sole stroke that does not contemplate the arm-pushing phase. Instead, it is the lower body that is responsible for the active propulsive phase during the stroke. Moreover, it has been shown that the energy expenditure during butterfly and breaststroke swimming is approximately twofold greater than in backstroke or front-crawl swimming [34]. Again, this was due to the increase in form drag dictated by the mechanics of these strokes. However, despite both butterfly and breaststroke sharing a symmetrical movement pattern, the breaststroke was shown to be the least efficient stroke in terms of energy expenditure and general in-water velocity. In fact, Pyne et al. [34] observed that the front crawl presented the lowest energy cost (1.23 kJ/m^−1^), followed by backstroke (1.47 kJ/m^−1^), butterfly (1.55 kJ/m^−1^), and breaststroke (1.87 kJ/m^−1^). Moreover, the swimming energy cost increased exponentially with an increase in swim velocity during freestyle, backstroke, and butterfly, but this change was linear in breaststroke [34].

In this regard, our findings transpose the in-water biomechanical disadvantages of breaststroke specialists into dry-land bio-motor disparities. The apparent bio-motor limitations on dry-land, the higher complexity of neuromuscular coordination between upper and lower limbs, as well as the inferior mechanical efficiency, put breaststroke in a unique as well as critical position regarding specific performance evaluation and improvement. All of the evidence considered, it may be that breaststroke performers depend more on maximizing their technical ability instead of their force–velocity production in a vertical pulling motion. In addition, given the major involvement of the lower body in generating propulsive forces during breaststroke, it is possible that the different contributions of the legs would be reflected in different force–velocity exertions between breaststroke and the other three strokes. In particular, we would suggest testing this hypothesis using either the back squat or bodyweight vertical jumps (e.g., countermovement jump), which are the most used and effective motions for indirectly improving the “lower-body-focused” elements of swimming races (i.e., diving and turning) [11,18,21].

This study is not exempt from limitations. Namely, the subjects enrolled had very specific characteristics regarding their competitive level (regional), training experience (6.5 ± 1.1 years of experience), and gender (male). On the one hand, the sample homogeneity allowed us to thoroughly analyze and compare several aspects of maximum force–velocity exertion and swimming performances. On the other hand, we cannot state if the findings from the present study would be confirmed either in athletes competing at the national/international level, holding more years of experience, or considering a population of female swimmers. Moreover, we only recruited swimmers specialized in a single stroke; however, swimmers can often compete over multiple specialties or medleys. What would the force–velocity capacities of this multi-specialized athlete be like? Perhaps, the higher grade of cross-training among strokes could bring some sort of technical/bio-motor transfer, which trainers should purposely take advantage of in order to improve specific aspects of a single stroke. However, this speculation needs to be verified with apt experimental designs investigating possible changes in swimmers’ maximum force–velocity exertion due to multifaceted training–competitive approaches.

## 5. Conclusions

Measuring maximum force–velocity exertion with the single pull-up test in regional-level swimmers may be a plain, scalable, lab-independent, cost-effective, and time-efficient experimental approach, apparently capable of discerning different levels of neuromuscular abilities based on stroke and distance specialization. However, it is debatable whether the results provided in this study are indeed a manifestation of different degrees of force–velocity capacities among distinctive categories of specialized swimmers, especially between sprinters and middle-distance swimmers, and between breaststroke and the other strokes. Therefore, we encourage continued investigation into this topic, to inform the process of developing evidence-based recommendations for scholars and trainers interested in enhancing swimming performance.

Finally, other sports could benefit from the evaluation of maximum force–velocity exertion for performance prediction and differentiation, especially closed-skill ones (as in swimming). This is because these kinds of sporting activities present almost no peer interactions and few environmental elements capable of affecting athletic performance, thus conceding sheer bio-motor abilities with considerable clout on the competitive outcome. However, it is also worth considering that swimming possesses many environmental elements that can affect performance and that differentiate it from other sports that are practiced on land in contrast to water. For these reasons, while the framework we proposed in this article could be incorporated within other sporting environments, it should also be rearranged for the specific sporting activity, with the ultimate goal of assessing and optimizing athletic performance for competitive endeavors.

## Figures and Tables

**Table 1:** Descriptive table of results.

		Velocity (m/s)			Force (N)		
Group	T (s)	Pull-Up Pre	Pull-Up Post	Fatigue (%)	Pull-Up Pre	Pull-Up Post	Fatigue (%)
Bu 50	27.35 ± 1.14	0.97 ± 0.07	0.95 ± 0.07	−2.33	1263.62 ± 161.23	1223.97 ± 155.62	−3.09
Ba 50	29.88 ± 0.96	0.95 ± 0.06	0.93 ± 0.06	−2.5	1230.66 ± 116.82	1194.58 ± 111.24	−2.88
Br 50	32.19 ± 0.89	0.79 ± 0.03	0.76 ± 0.03	−2.86	1047.83 ± 61.33	1016.11 ± 66.81	−2.95
FC 50	24.86 ± 0.87	0.96 ± 0.06	0.94 ± 0.05	−2.15	1247.48 ± 137.14	1211.46 ± 140.39	−2.85
Bu 100	60.49 ± 1.16	0.88 ± 0.02	0.86 ± 0.02	−2.27	1175.36 ± 53.48	1146.21 ± 55.09	−2.45
Ba 100	63.82 ± 2.32	0.88 ± 0.05	0.86 ± 0.05	−1.71	1151.48 ± 31.28	1111.98 ± 29.45	−3.39
Br 100	71.72 ± 2.19	0.77 ± 0.03	0.75 ± 0.03	−2.25	1024.42 ± 29.51	999.12 ± 26.57	−2.43
FC 100	53.63 ± 1.18	0.93 ± 0.04	0.92 ± 0.03	−1.72	1188.69 ± 55.30	1156.16 ± 58.93	−2.68
Bu 200	151.16 ± 13.33	0.66 ± 0.03	0.65 ± 0.04	−2.28	963.63 ± 28.62	925.44 ± 31.56	−3.9
Ba 200	144.79 ± 8.13	0.67 ± 0.03	0.65 ± 0.03	−2.61	960.82 ± 29.90	930.14 ± 28.98	−3.11
Br 200	158.45 ± 4.49	0.67 ± 0.03	0.66 ± 0.03	−2.24	957.55 ± 34.13	932.50 ± 36.60	−2.56
FC 200	120.63 ± 4.71	0.69 ± 0.04	0.67 ± 0.04	−2.52	966.85 ± 33.89	940.01 ± 27.95	−2.69

Abbreviations: T = time of the swimming race; Bu = butterfly; Ba = backstroke; Br = breaststroke; FC = front crawl; fatigue (%) = percentage change between pull-up pre and pull-up post the swimming race. The numbers “50”, “100”, and “200” next to each group indicate the distances in which the swimmers specialized.

**Table 2:** Multiple linear regression analysis to predict swimming performances based on z-scored velocity and force generated in the single pull-up test. The analysis considers both the distance and stroke groups.

**ANOVA**					
**Model**	**DF**	**Sum of Square**	**Mean Square**	**F Statistic**	***p*-Value**
Regression	2	33.77	16.88	78.17	<0.001
Residual	93	50.09	0.54		
Total	95	83.86	0.88		
**Coefficients (R-square = 0.81; adjusted R-squared = 0.80)**					
**Model**	**Estimate**	**Standard Error**	**t-Value**	***p*-Value**	
(Constant)	3.03 × 10^−14^	0.075	1.08	0.28	
Velocity (z-score)	−0.046	0.126	−3.90	<0.001	
Force (z-score)	−0.037	0.121	−3.60	<0.001	

**Table 3:** ANOVA tables for velocity (m/s) and force (N) in the single pull-up test before the swimming competition.

**Velocity (m/s)**					
**Source**	**DF**	**Sum of Square**	**Mean Square**	**F Statistic**	***p*-Value**
Groups (between groups)	11	1.36	0.12	66.80	<0.001
Error (within groups)	84	0.16	0.0019		
Total	95	1.52	0.016		
**Force (N)**					
**Source**	**DF**	**Sum of Square**	**Mean Square**	**F Statistic**	***p*-Value**
Groups (between groups)	11	1,329,427.64	120,857.06	19.60	<0.001
Error (within groups)	84	517,981.89	6166.45		
Total	95	1,847,409.53	19,446.42		

**Table 4:** Bonferroni post-hoc comparisons test for velocity (m/s) and force (N) across the experimental groups. The mean differences are shown. The asterisk * shows that the mean difference is significant at the 0.05 level. Interestingly, most of the significant post-hoc differences in velocity corresponded to the same significant post-hoc differences in force among the experimental groups, further strengthening the suggestion that the velocity and force generated executing a vertical pulling motion are (almost) linearly intertwined.

**Velocity (m/s)**											
**Group**	**Ba 50**	**Br 50**	**FC 50**	**Bu 100**	**Ba 100**	**Br 100**	**FC 100**	**Bu 200**	**Ba 200**	**Br 200**	**FC 200**
Bu 50	0.02	0.20 *	0.001	0.10	0.10	0.20 *	0.04	0.31 *	0.30 *	0.30 *	0.28 *
Ba 50	0	0.17 *	0.01	0.08	0.08	0.18 *	0.02	0.29 *	0.29 *	0.28 *	0.26 *
Br 50	0.17 *	0	0.18 *	0.11 *	0.10 *	0.02	0.15 *	0.12 *	0.18 *	0.11 *	0.11 *
FC 50	0.01	0.18 *	0	0.09	0.09	0.19 *	0.03	0.30 *	0.30 *	0.29 *	0.27 *
Bu 100	0.08	0.10	0.09	0	0.004	0.11 *	0.05	0.22 *	0.21 *	0.21 *	0.19 *
Ba 100	0.08	0.09	0.08	0.003	0	0.11 *	0.05	0.21 *	0.21 *	0.20 *	0.19 *
Br 100	0.18 *	0.01	0.19 *	0.11 *	0.11 *	0	0.16 *	0.11 *	0.11 *	0.12 *	0.12 *
FC 100	0.02	0.15	0.03	0.05	0.06	0.16 *	0	0.27 *	0.27 *	0.26 *	0.24 *
Bu 200	0.29 *	0.10 *	0.30 *	0.22 *	0.21 *	0.11 *	0.27 *	0	0.003	0.08	0.03
Ba 200	0.29 *	0.10 *	0.30 *	0.21 *	0.21 *	0.10 *	0.27 *	0.003	0	0.07	0.03
Br 200	0.28 *	0.09 *	0.29 *	0.21 *	0.20 *	0.10 *	0.26 *	0.01	0.01	0	0.02
**Force (N)**											
**Group**	**Ba 50**	**Br 50**	**FC 50**	**Bu 100**	**Ba 100**	**Br 100**	**FC 100**	**Bu 200**	**Ba 200**	**Br 200**	**FC 200**
Bu 50	32.96	215.79 *	16.14	88.26	112.13	239.21 *	74.93	299.99 *	302.80 *	306.07 *	296.77 *
Ba 50	0	182.83 *	16.82	55.30	79.18	206.25 *	41.97	267.04 *	269.84 *	273.11 *	263.81 *
Br 50	182.83 *	0	199.65 *	127.54 *	103.66 *	23.42	140.86 *	84.20 *	87.01	90.28 *	80.98 *
FC 50	16.82	199.65 *	0	72.12	96	223.07 *	58.79	283.86 *	286.66 *	289.93 *	280.63 *
Bu 100	55.30	127.54	72.12	0	23.88	150.95 *	13.33	211.74 *	214.54 *	217.82 *	208.63 *
Ba 100	79.18	103.66	96	23.88	0	127.07 *	37.20	187.86 *	190.67 *	193.94 *	184.64 *
Br 100	206.25 *	23.42	223.07 *	150.95 *	127.07 *	0	164.28 *	60.79 *	63.59 *	66.86 *	57.56 *
FC 100	41.97	140.86 *	58.79	13.33	37.20	164.28 *	0	225.06 *	227.87 *	231.14 *	221.84 *
Bu 200	267.04 *	84.20 *	283.86 *	211.74 *	187.86 *	60.79 *	225.06 *	0	2.81	6.08	3.22
Ba 200	269.84 *	87.01 *	286.66 *	214.54 *	190.67 *	63.59 *	227.87 *	2.81	0	3.27	6.03
Br 200	273.11 *	90.28 *	289.93 *	217.82 *	193.94*	66.86 *	231.14 *	6.08	3.27	0	9.3

Abbreviations: Bu = butterfly; Ba = backstroke; Br = breaststroke; FC = front crawl. The asterisk * shows that the mean difference is significant at the 0.05 level.

## Data Availability

Data are available on request.
